# The Impact of the Position of the Upper Eyelid on the Profile of Epithelial Thickness and the Topography of the Cornea

**DOI:** 10.3390/jcm14041327

**Published:** 2025-02-17

**Authors:** Kroczek Marta, Kudelska Dagmara, Młyniuk Patryk, Kałużny Bartłomiej

**Affiliations:** 1 Division of Ophthalmology and Optometry, Department of Ophthalmology, Collegium Medicum, Nicolaus Copernicus University, 85-067 Bydgoszcz, Poland; 2 Oftalmika Eye Hospital, 85-631 Bydgoszcz, Poland

**Keywords:** ptosis, upper eyelid, elevation map, epithelial thickness, corneal aberrations

## Abstract

**Aim**: To evaluate the impact of the position of the upper eyelid on the corneal epithelial thickness, anterior elevation map, and corneal aberrations in patients with unimpaired function of the eyelids. **Methods:** Sixty-one right eyes were included in this prospective, non-randomized study. The low-positioned eyelid group (LP group) consisted of 30 patients with a mean upper eyelid margin position at 3.45 ± 0.45 mm above the corneal apex at primary gaze. The high-positioned eyelid group (HP group) comprised 31 eyes for which the respective value was 4.56 ± 0.36 mm. The anterior elevation map, corneal aberrations, and epithelial thickness profile were obtained with eyes wide open, using a MS-39 corneal tomograph (CSO, Florence, Italy). The analysis was also performed at measurement points 0.3 mm above and 0.3 mm below the eyelid margin. **Results**: Significantly thinner epitheliums at 4.5 mm above the center of the cornea on the vertical meridian in the LP group were observed (*p* < 0.05). Higher anterior elevations and thicker epitheliums of the cornea were observed at the measurement location of 0.3 mm above the upper eyelid’s margin in the HP group (*p* < 0.05). A negative correlation between the height of the upper eyelid and spherical aberration was noted. **Conclusions**: The findings provide evidence that the positioning of the upper eyelid in individuals with normal eyelid function influences the epithelial thickness map, the anterior elevation map, and corneal spherical aberrations.

## 1. Introduction

Corneal topography and tomography play a fundamental role in the qualitative assessment of the cornea [1]. The utilization of topography based on Placido disks modern devices enables the measurement of anterior surface curvatures, including the central, paracentral, and peripheral cornea [2]. This involves projecting alternating light and dark rings from the Placido disk onto the corneal surface, followed by analysis of their reflections [3,4]. Despite the limitation of being unable to image the posterior surface of the cornea, Placido disk topography is commonly used for diagnosing corneal ectasia, planning refractive surgeries, and fitting contact lenses [5,6]. Tomography, conversely, is utilized for the assessment of corneal morphology and morphometry through the acquisition of three-dimensional representations of the anterior and posterior surfaces and is capable of providing corneal thickness maps [3,7]. Various tomography imaging techniques such as slit scanning technology, very-high frequency scanning, rotational Scheimpflug camera, and optical coherence tomography are used for this purpose [5]. Optical coherence tomography (OCT) utilizes low-coherence interferometry to create cross-sectional images by registering infrared light scattered back from structures in the anterior segment and comparing it with a reference reflection [7,8]. Additional measurements such as anterior chamber depth, lens thickness, scleral spur angle, and pupil diameter can also be obtained using OCT [9]. The mapping of anterior and posterior curvature or thickness of the cornea through non-invasive tools contributes significantly to the diagnosis of early stages of keratoconus and the identification of patients at high risk of postoperative ectasia following procedures like laser in situ keratomileusis (LASIK) [9,10,11]. One of the more recent advancements pertains to the capability of quantifying and mapping the thickness of the corneal epithelium. This advancement allows for a more comprehensive evaluation of corneal health and pathology, as well as aids in the customization of treatment plans for patients with corneal irregularities [12].

There is long-standing evidence indicating that the position of the lids influences corneal topography [13,14]. Previous studies were conducted using Placido disk topography, and there are some papers suggesting that there is a difference in the topography measurements with and without the lids touching the corneal surface [15,16]. The first studies about the influence of eyelids forces on the corneal epithelium were published in the 1980-/1990s and investigated the epithelial morphology in keratoconjuctivitis sicca, contact lens wearing, and neurotrophic keratitis and also examined in vitro rabbit eyes [17,18]. Subsequent studies investigated how the position of the upper eyelid affected corneal parameters in individuals with eyelid abnormalities like ptosis or blepharospasm [19,20,21,22,23,24]. Findings from these studies indicate that differences in the upper eyelid position within such individuals may result in variations in the distribution of epithelial thickness, consequently affecting corneal topography. Researchers have inferred that the pressure exerted by the tarsus contributes to alterations in epithelial thickness, thereby influencing the shape of the anterior corneal surface. Our research aims to validate, for the first time, the hypothesis that the position of the upper eyelid, which is a subject of population variability, significantly impacts the profile of epithelial thickness and corneal topography in individuals with unimpaired function of the eyelids.

## 2. Material and Methods

### 2.1. Material

This prospective, non-randomized study was performed in Bydgoszcz, Poland, and included 61 Caucasian patients. Patient mean age was 34 ± 14.23 years. The study group consisted of 37 men and 24 women. The study was conducted in accordance with the Declaration of Helsinki and was approved by the Ethics Committee in Bydgoszcz, Poland (KB 502/2022, approved on 25 October 2022). All patients provided written informed consent before the initiation of any study-related procedures. 

### 2.2. Study Design

Only the right eyes of 61 patients were enrolled in the study. The inclusion criterion required that, in primary gaze with the eye naturally open, the upper eyelid covered the corneal limbus at the 12 o’clock position. Exclusion criteria included patients with ocular surface diseases, meibomian gland dysfunction, other eyelid abnormalities, or contact lens use. Patients whose corneal limbus was covered by the lower eyelid were also excluded.

The MS-39 corneal tomograph (CSO, Florence, Italy) was used for the study. The high-resolution tool combining Placido disk topography and spectral-domain optical coherence tomography (SD-OCT) provides a transversal resolution of 35 nm in air and an axial resolution of 3.6 mm in tissue and uses a wavelength of 845 nm [19]. The light source used in the tool is a super-luminescent light-emitting diode (SLED) [2]. The device captures on average, one keratoscopic scan, 25 SOCT scans, and one iris front image in 1 s [20].

**Figure 1:**
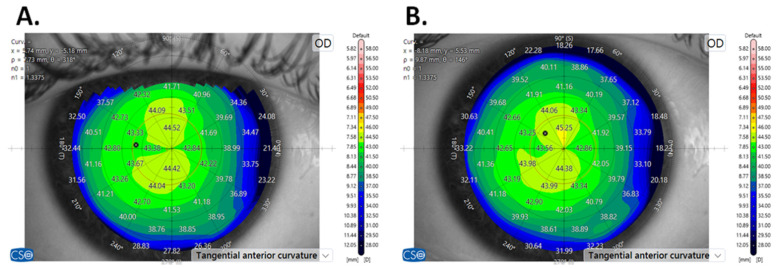
Corneal tomography imaging with palpebral fissure with naturally opened eyes (**A**) and with eyes widely opened (**B**).

Patients were divided into two groups depending on the height of the upper eyelid of naturally opened eyes at the primary gaze position. The measurement was taken from the corneal vertex to the upper eyelid margin in the vertical meridian, thus corresponding to the Margin Reflex Distance-1 (MRD-1), as the corneal light reflex aligns with the corneal vertex [21]. MS-39 was used to indicate how the position of the upper eyelid impacts corneal topography. Topographic scans and anterior eye images were performed in primary gaze with naturally opened eyes (Figure 1A) to evaluate the upper lid position. Subsequently, scans were repeated with eyes forcefully opened wider than their natural position (Figure 1B) to obtain maps covering the entire anterior corneal surface. The high-positioned group included 31 eyes with heights between 4.11 and 5.58 mm (mean 4.56 ± 0.36 mm) and the low-positioned group consisted of 30 eyes with heights between 2.21 and 4.08 mm (mean 3.45 ± 0.45 mm). The basic parameters of the study groups are presented in Table 1.

**Figure 2:**
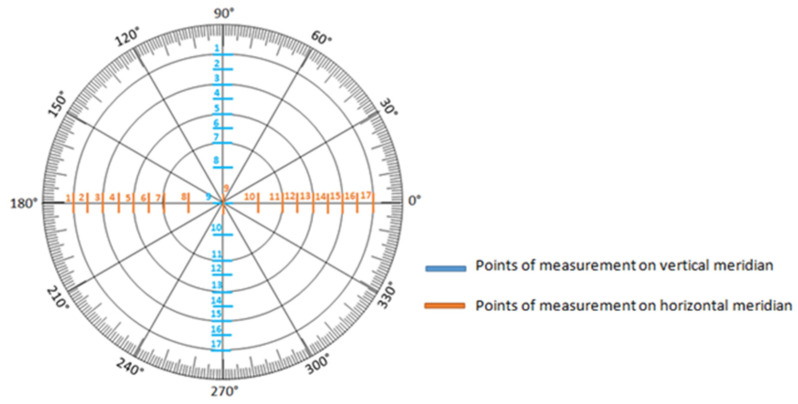
Measurement points of anterior elevation map and epithelial thickness map on relevant maps.

Anterior elevation maps and epithelial thickness maps over a surface area with a diameter of 9 mm were measured. Epithelial thickness was measured on the vertical (90 degrees) and horizontal (180 degrees) meridians at 17 regularly arranged points (Figure 2). Anterior elevation maps were also measured at the same points. To evaluate indirectly the influence of the upper eyelid compression on the cornea, the measurements were performed at a distance of 0.3 mm above and 0.3 mm below the eyelid’s margin. In this study the following factors were investigated: total optical path difference (OPD)—the total amount of the wavefront error within the analysis diameter of 5 mm;−higher orders—the amount of the wavefront corresponding to the order of polynomials from 3 to 7;−coma aberrations—the amount of polynomials Z_3_^−1^ and Z_3_^+1^_;_−astigmatism—the amount of polynomials Z_2_^−2^ and Z_2_^+2^;−spherical aberrations—the amount of polynomial Z_4_^0^; and residual—the total amount of the wavefront removing the number of coma, astigmatism, and spherical aberrations.

### 2.3. Statistical Analysis

Statistics 26 and IBM SPSS Statistics (Armonk, NY, USA) were used for the analysis of the collected data. The Shapiro-Wilk test was used to determine the normality of variable distribution. For all comparisons, the level of statistical significance was *p* = 0.05. Summary statistics for variables with normal distribution are presented in the study by mean value (M) and standard deviation (SD). The Student *t*-test for independent groups and The Mann-Withney test were used to identify differences between groups. Descriptive statistics, and Pearson, and Spearman correlation coefficients were also used.

## 3. Results

**Figure 3:**
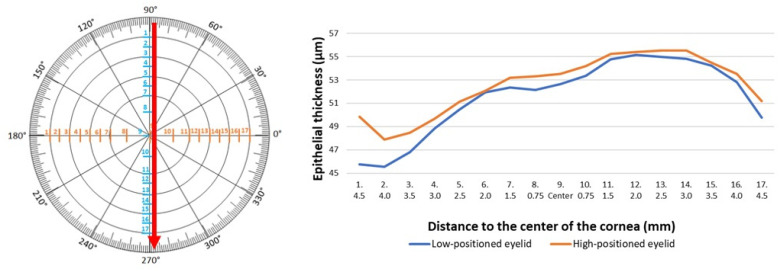
Epithelial thickness measured vertically in patients with the low- and high-positioned upper eyelids.

**Figure 4:**
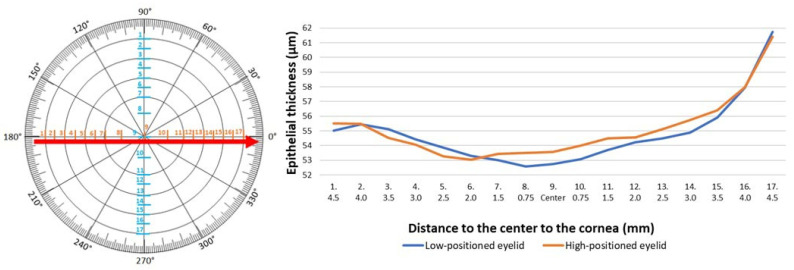
Epithelial thickness measured horizontally in patients with the low− and high-positioned upper eyelids.

The comparative analysis of epithelial thickness in two groups was conducted. The study showed differences in the epithelial thickness in the low- and high-positioned upper eyelids. A statistically significant difference was observed 4.5 mm above the center of the cornea on the vertical meridian, where the epithelial thickness was significantly thinner in the cases of the low-positioned upper eyelids compared to the high-positioned eyelids (*p* = 0.01) (Table 2, Figure 3). Measurements of epithelial thickness on the horizontal meridian showed no significant differences. Despite the position of the upper eyelid, the thickness of corneal epithelium was similar in 17 measurement points (Table 3, Figure 4).

**Figure 5:**
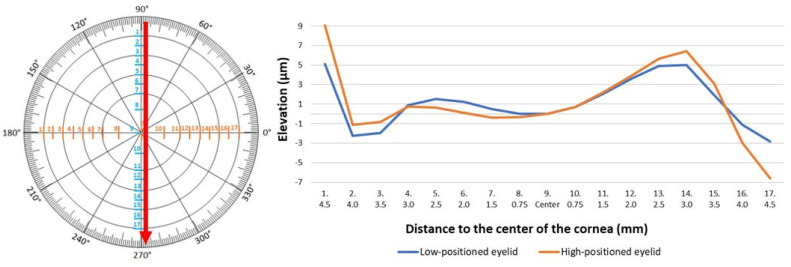
Elevation in the anterior part of the cornea measured vertically in low- and high-positioned upper eyelids.

**Figure 6:**
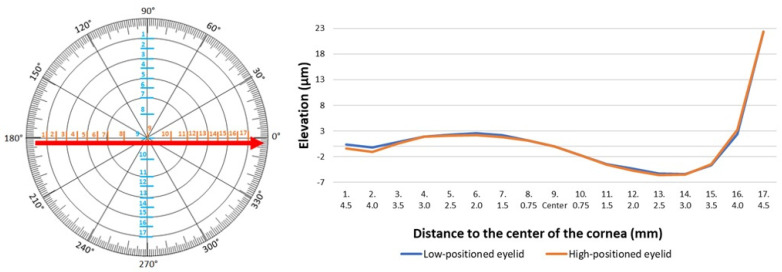
Anterior elevation of the cornea measured horizontally in low- and high-positioned upper eyelids.

Anterior elevation map was analyzed at the same 17 measurement points. The study did not reveal any crucial differences in the elevation of the anterior surface of the cornea. Results in the two groups were similar both on the vertical and horizontal meridians (Table 4 and Table 5; Figure 5 and Figure 6).

The second part of this analysis aimed to compare measurements conducted 0.3 mm above and below the upper eyelid’s margin in patients with high- and low-positioned upper eyelids. The analysis revealed a significantly greater thickness of epithelium at a measurement location 0.3 mm above the eyelid margin for patients with high-positioned eyelids in comparison to patients with low-positioned eyelids (*p* = 0.009) (Table 6). The anterior elevation map measurements were examined at the same points. Thise study shows the statistical significance of elevation in the anterior surface of the cornea 0.3 mm above the eyelid margin (*p* < 0.01). Patients with high-positioned upper eyelids had significantly higher elevation compared to patients with low-positioned upper eyelids.

A comparison of the study groups showed no significant differences in corneal aberrations (Table 7). However, the Spearman rank correlation coefficient showed a significant but weak negative correlation between the height of the eyelid and the quantity of spherical aberrations (*p* = 0.044) (Table 8).

## 4. Discussion

This study verified, for the first time, the hypothesis that the upper eyelid’s position has an impact on epithelial thickness profile and corneal topography in patients with unimpaired function of the eyelids. Epithelial thickness in patients with high-positioned upper eyelids is significantly thicker at 4.5 mm above the center of the cornea compared to that in patients with low-positioned eyelids. The result suggested that the upper eyelid generates compression extended on the corneal surface which causes a decrease in epithelial thickness. The corneal epithelium measured on the horizontal meridian had a similar thickness independently of the upper eyelid’s position. Differences in the anterior elevation map based on the upper eyelid height were found to lack statistical significance. Nevertheless, the analysis of the impact of the eyelid’s position on the anterior elevation map showed a significantly higher elevation 0.3 mm above the eyelid margin in the high-positioned eyelid. The epithelial thickness at this measurement point was also significantly higher. A comparison of epithelial thickness and elevation on the vertical meridian for the same parts of the cornea showed one statistically significant difference, but measurements analyzed at equal distance from eyelid margin seems to have a better correlation with the actual position of eyelid.

In our study, a negative correlation was observed between the height of the upper eyelid and spherical aberrations. The correlation was statistically significant but weak. The quantity of other aberrations was not dependent on the height of the upper eyelid.

In the published literature, there are studies on eyelid forces as far back as the 1980s. Lemp and Mathers proposed a hypothesis that eyelid-induced shearing forces enhances epithelial cell turnover and exfoliation [17]. An in vitro experiment with rabbit eyes supported that hypothesis and showed that shear force on the corneal epithelium significantly increases cell shedding, with six times more cells removed from shear-treated corneas compared to static ones over a period of 4 h [18]. 

Reinstein, D.Z., et al. investigated epithelial thickness measurements by using high-frequency digital ultrasound signal processing already in 1994 [12]. In 2008, using Artemis 1 (Ultralink LCC, Gauteng, South Africa), a very high-frequency digital ultrasound scanner, it was demonstrated that the corneal epithelial thickness is diversified. In the superior half of the cornea, the epithelial thickness is reduced compared to the inferior half, and in the temporal region of the cornea, the epithelium in thinner compared to the nasal region [22]. It is possible that eyelid forces and blinking action could have a significant impact on the epithelial thickness profile. The upper eyelid’s friction against the surface of the cornea during blinking creates a template for the outer shape of the epithelial surface.

In 1978, Kiely and Carney proposed that the position of the lids influences corneal topography. Nevertheless, during that era, diagnostic capabilities were quite restricted and measurements of corneal epithelial thickness were not feasible [14]. Several, more recent studies evaluated epithelial thickness changes and corneal aberrations in different abnormalities of the upper eyelid, such as ptosis or blepharospasm [19,20,21,22,23,24]. Zhang et al. measured the effects of blepharospasm on the cornea using a Pentacam rotating Scheimpflug camera (Oculus, Inc., Wetzlar, Germany). The study showed significantly higher keratometric parameters (for example, anterior flat keratometry (K1), and mean keratometry (Km)) in moderate and severe blepharospasm in comparison to the control group [23]. Corneal elevation parameters did not differ significantly from patients in the control group except for the front BFS (Best Fit Sphere) diameter, which was significantly lower in patients with blepharospasm. The results support the hypothesis that corneal shape changes can be caused by the redistribution of upper eyelid pressure in patients with blepharospasm.

The same Pentacam rotating Scheimpflug camera system was used to create topographic and tomographic scans of Chinese patients with congenital ptosis in comparison to a control group with a normal eyelid position. Shen et al. demonstrated that the values of K1, Km, and steep posterior keratometry (K2) were lower than in patients in the control group. Similarly, in the case of ptosis, the corneal thickness and thinnest point of the cornea were significantly lower [20]. In the study conducted by Bhattacharjee et al., high-order aberrations and corneal topography were analyzed before and after the upper eyelid blepharoplasty surgery [19]. The measurements were obtained by using Topographic Modelling System (TMS-4) (Tomey Corporation Japan, Nagoya, Japan). The authors argue that the magnitude of high-order aberrations decreases after surgery. In the topographic scans, only the cylinder value changed significantly (from a mean of 0.68 to 0.48 D). Dogan et al. analyzed corneal epithelial thickness in cases of congenital myogenic eyelid ptosis compared to the normally positioned upper eyelid using anterior segment optical coherence tomography (AS-OCT) scans. The study showed significantly thinner epithelium in the superior sectors compared to the inferior part of the cornea. Changes in the corneal epithelial thickness can be observed as a result of the mechanical effect of the ptotic eyelid on the surface of the cornea [24].

The impact of dermatochalasis and upper eyelid blepharoplasty surgery on epithelial thickness was also evaluated using AS-OCT measurements. Arslan et al. showed significant differences in epithelial thickness between patients with dermatochalasis and the control group. Six months after surgery, the thickness of the epithelium increased significantly compared to patients before blepharoplasty surgery [25]. Carreira et al. showed a significant difference between epithelial thickness in superior and inferior octants of the cornea before upper eyelid blepharoplasty surgery in comparison to the measurements obtained after surgery by using Pentacam and Anterior segment optical coherence tomography (AS-OCT) (Zeiss Cirrus 5000 HD-OCT, Oberkochen, Germany). The results suggest that pressure of the tarsus leads to epithelial thickness changes and, as a result, modifies steep corneal keratometry [26].

In conclusion, the position of the upper eyelid influences the corneal epithelial thickness, anterior elevation map, and corneal spherical aberrations. The pressure exerted by the tarsus contributes to the alterations to these parameters not only in eyes with eyelid abnormalities, like ptosis or blepharospasm, but also in eyes with the unimpaired function of the eyelids.

## Figures and Tables

**Table 1:** Characteristics of study groups; mean (SD).

	Low-Positioned Eyelid n = 30	High-Positioned Eyelid n = 31	*p*
Height of upper eyelid, mm	3.45 (0.45)	4.56 (0.36)	**<0.001**
Spherical error, D	−0.26 (0.92)	−0.32 (0.85)	0.8
Cylindrical error, D	−0.21 (0.38)	−0.23 (0.34)	0.87
Spherical equivalent, D	−0.36 (1.02)	−0.43 (0.89)	0.79
Thk min, µm	546.48 (32.34)	542.67 (29.17)	0.63
Epi-Thk min, µm	47.58 (4.38)	47.73 (4.68)	0.9
Keratometry—flat meridian, D	43.41 (1.72)	43.0 (1.28)	0.35
Keratometry—steep meridian, D	44.3 (1.83)	43.95 (1.42)	0.5
Keratometry average, D	43.85 (1.76)	43.45 (1.33)	0.41
Keratometry cylinder, D	−0.81 (0.43)	−0.97 (0.45)	0.15
W-W	11.72 (0.43)	11.77 (0.23)	0.93
Age, years	35.68 (14.46)	30.7 (11.40)	
Sex distribution—Male, %	8 (26.67) *	12 (38.71) *	
Sex distribution—Female, %	22 (73.33) *	19 (61.29) *	

* The value is number of male/female patients in the group (percentage of patients in the group).

**Table 2:** Comparison of epithelial thickness on the vertical meridian in two study groups.

Distance to the Center of the Cornea in Vertical Meridian (mm)	Low-Positioned Eyelid	High-Positioned Eyelid	*p*
	Mean [µm] (SD) n = 30	Mean [µm] (SD) n = 31	
4.5 above	45.74 (5.95)	49.83 (6.10)	**0.010**
4.0 above	45.55 (4.57)	47.90 (4.94)	0.058
3.5 above	46.81 (4.33)	48.47 (4.38)	0.142
3.0 above	48.84 (4.49)	49.70 (4.8)	0.471
2.5 above	50.48 (4.02)	51.13 (5.03)	0.579
2.0 above	51.94 (3.32)	52.07 (4.94)	0.903
1.5 above	52.35 (3.09)	53.17 (4.54)	0.416
0.75 above	52.13 (3.18)	53.30 (4.41)	0.238
Center	52.65 (3.06)	53.53 (4.24)	0.351
0.75 below	53.35 (2.68)	54.17 (4.31)	0.383
1.5 below	54.77(2.39)	55.23 (4.34)	0.613
2.0 below	55.13 (2.53)	55.40 (3.94)	0.752
2.5 below	54.97 (2.70)	55.53 (4.07)	0.523
3.0 below	54.81 (3.48)	55.53 (3.69)	0.432
3.5 below	54.23 (3.82)	54.47 (3.79)	0.805
4.0 below	52.81 (4.50)	53.53 (3.90)	0.504
4.5 below	49.77 (6.43)	51.20 (4.24)	0.312

**Table 3:** Comparison of epithelial thickness on the horizontal meridian in two study groups.

Distance to the Center of the Cornea on the Horizontal Meridian [mm]	Low-Positioned Eyelid n = 30	High-Positioned Eyelid n = 31	*p*
	Mean [µm] (SD)	Mean [µm] (SD)	
4.5 nasally	55.03 (4.28)	55.50 (4.56)	0.681
4.0 nasally	55.45 (3.91)	55.47 (4.31)	0.989
3.5 nasally	55.13 (3.64)	54.53 (4.70)	0.581
3.0 nasally	54.42 (3.15)	54.07 (4.59)	0.727
2.5 nasally	53.87 (2.99)	53.27 (4.61)	0.544
2.0 nasally	53.32 (3.04)	53.03 (4.27)	0.761
1.5 nasally	53.00 (2.94)	53.43 (4.26)	0.645
0.75 nasally	52.58 (3.04)	53.50 (4.44)	0.348
Center	52.74 (3.00)	53.57 (4.20)	0.380
0.75 temporally	53.06 (3.03)	54.00 (4.38)	0.335
1.5 temporally	53.71 (2.69)	54.50 (4.16)	0.380
2.0 temporally	54.23 (2.93)	54.57 (3.74)	0.693
2.5 temporally	54.48 (2.86)	55.13 (3.79)	0.452
3.0 temporally	54.90 (3.00)	55.73 (3.78)	0.345
3.5 temporally	55.90 (3.43)	56.40 (3.89)	0.598
4.0 temporally	57.94 (3.65)	57.97 (4.54)	0.976
4.5 temporally	61.74 (4.70)	61.40 (6.09)	0.807

**Table 4:** Comparison of elevation of anterior surface on the vertical axis in two study groups.

Distance to the Center of the Cornea on the Vertical Meridian [mm]	Low-Positioned Eyelid n = 30	High-Positioned Eyelid n = 31	*p*
	Mean [µm] (SD)	Mean [µm] (SD)	
4.5 above	5.10 (11.92)	9.07 (15.95)	0.274
4.0 above	−2.26 (4.36)	−1.10 (3.82)	0.275
3.5 above	−1.97 (3.55)	−0.80 (3.42)	0.196
3.0 above	0.87 (5.18)	0.73 (3.72)	0.906
2.5 above	1.55 (4.46)	0.67 (3.56)	0.398
2.0 above	1.23 (4.06)	0.10 (3.09)	0.229
1.5 above	0.52 (3.05)	−0.37 (2.63)	0.232
0.75 above	0.03 (1.68)	−0.33 (1.56)	0.383
Center	0.00 (0.00)	0.00 (0.00)	-
0.75 below	0.71 (1.99)	0.70 (1.60)	0.983
1.5 below	2.06 (3.41)	2.20 (2.99)	0.870
2.0 below	3.58 (4.03)	3.87 (3.39)	0.766
2.5 below	4.90 (3.88)	5.63 (3.70)	0.456
3.0 below	5.00 (3.44)	6.43 (3.46)	0.110
3.5 below	1.90 (3.24)	3.10 (2.71)	0.123
4.0 below	−1.13 (5.88)	−2.97 (4.49)	0.176
4.5 below	−2.84 (9.05)	−6.60 (9.16)	0.112

**Table 5:** Comparison of elevation of anterior surface on the horizontal meridian in the two study groups.

Distance to the Center of the Cornea on the Horizontal Meridian [mm]	Low-Positioned Eyelid n = 30	High-Positioned Eyelid n = 31	*p*
	Mean [µm] (SD)	Mean [µm] (SD)	
4.5 nasally	0.32 (5.26)	−0.43 (4.70)	0.556
4.0 nasally	−0.23 (2.49)	−1.10 (3.14)	0.232
3.5 nasally	0.81 (2.46)	0.53 (2.26)	0.653
3.0 nasally	1.94 (2.99)	1.93 (2.33)	0.998
2.5 nasally	2.32 (3.03)	2.13 (2.26)	0.783
2.0 nasally	2.52 (2.83)	2.20 (1.90)	0.609
1.5 nasally	2.19 (2.15)	1.83 (1.58)	0.460
0.75 nasally	1.16 (1.07)	1.10 (0.88)	0.808
Center	0.00 (0.00)	0.00 (0.00)	-
0.75 temporally	−1.71 (1.30)	−1.80 (0.89)	0.753
1.5 temporally	−3.48 (2.54)	−3.57(1.43)	0.877
2.0 temporally	−4.39 (3.22)	−4.70 (1.78)	0.640
2.5 temporally	−5.32 (3.70)	−5.57 (2.16)	0.753
3.0 temporally	−5.39 (3.66)	−5.50 (2.42)	0.887
3.5 temporally	−3.71 (2.89)	−3.47 (3.01)	0.749
4.0 temporally	2.42 (3.92)	3.27 (3.42)	0.373
4.5 temporally	22.35 (10.86)	22.33 (7.68)	0.993

**Table 6:** Epithelial thickness and elevation of anterior corneal surface 0.3 mm above and 0.3 mm below the lid margin in two study groups.

	Low-Positioned Eyelid n = 30	High-Positioned Eyelid n = 31	*p*
	Mean (SD)	Mean (SD)	
Epithelial thickness 0.3 mm above the lid margin [µm]	45.87 (5.00)	50.23 (7.46)	**0.009**
Epithelial thickness 0.3 mm below the lid margin [µm]	47.77 (4.49)	48.90 (4.44)	0.329
Front elevation 0.3 mm above the lid margin [µm]	−1.77 (5.28)	20.57 (27.91)	**<0.001**
Front elevation 0.3 mm below the lid margin [µm]	−0.26 (4.28)	4.87 (13.90)	0.062

**Table 7:** Corneal aberrations in two study groups.

Type of Aberrations	Low-Positioned Eyelid n = 30		High-Positioned Eyelid n = 31		
	M [µm]	SD	M [µm]	SD	*p*
Astigmatism	0.38	0.21	0.47	0.29	0.180
Coma aberrations	0.21	0.12	0.19	0.13	0.186
Spherical aberrations	0.20	0.19	0.20	0.22	0.154
Residual aberrations	0.16	0.06	0.16	0.05	0.627
Optical path difference (OPD)	0.44	0.24	0.44	0.27	0.974
High order aberrations (HoA)	0.28	0.14	0.26	0.11	0.533

**Table 8:** Correlation between height of the upper eyelid and corneal aberrations.

Type of Aberrations	Variable	Type of Correlation Coefficient	Result	*p*
Astigmatism	Height of upper eyelid	Pearson	0.11	0.403
Coma aberrations		Spearman’s Rho	−0.18	0.73
Spherical aberrations		Spearman’s Rho	**−0.26**	**0.044**
Residual aberrations		Pearson	−0.05	0.678
Optical path difference (OPD)		Pearson	−0.05	0.730
High−order Aberrations (HoA)		Pearson	−0.21	0.098

## Data Availability

The original contributions presented in this study are included in the article. Further inquiries can be directed to the corresponding author.
